# Secondary metabolite credentials of *Evolvulus alsinoides* by high performance thin layer chromatography (HPTLC)

**DOI:** 10.7555/JBR.26.20110128

**Published:** 2012-07-07

**Authors:** Duraisamy Gomathi, Manokaran Kalaiselvi, Ganesan Ravikumar, Dominic Sophia, Velliyur Kanniappan Gopalakrishnan, Chandrasekar Uma

**Affiliations:** Department of Biochemistry, Karpagam University, Coimbatore 641021, India.

**Keywords:** *Evolvulus alsinoides*, high performance thin layer chromatography (HPTLC), secondary metabolites, convolvulaceae

## Abstract

Plants and plant-based products are the bases of many modern pharmaceuticals that are current in use today for various diseases. The aim of the study was to investigate the biochemical constituents and high performance thin layer chromatography (HPTLC) finger printing of the ethanolic extract of *Evolvulus alsinoides*. Phytochemical screening was done by standard procedures and HPTLC method was also established to analyze alkaloids, flavonoids and phenolic compounds from the ethanolic extract of *Evolvulus alsinoides*. Preliminary phytochemical screening showed that ethanol extracted more secondary metabolites than other solvents. HPTLC fingerprinting analysis showed the presence of various alkaloids, flavonoids and phenols (quercetin) in the ethanolic extract. It can be concluded that *Evolvulus alsinoides* may serve as a source of potent antioxidants that may be used in the prevention of various diseases such as cancer, diabetes and cardiovascular diseases due to the presence of phenolic compounds. HPTLC finger print of *Evolvulus alsinoides* may be useful in the differentiation of the species from adulterants and act as a biochemical marker for this medicinally important plant in the pharmaceutical industry and plant systematic studies.

## INTRODUCTION

Plants have been an important source of medicines for thousands of years. Even today, the World Health Organization (WHO) estimates that up to 80 percent of people still rely mainly on traditional remedies such as herbs for their medicines[1]-[3]. Herbs are now very popular in developing countries due to improved knowledge about the safety, efficacy and quality assurance of ethnomedicine. In recent years, secondary plant metabolites (phytochemicals) have been extensively investigated as a source of medicinal agents. Thus, it is anticipated that phytochemicals with good antibacterial activity will be used for the treatment of bacterial infections[4],[5].

*Evolvulus alsinoides* (*E. alsinoides*) is a perennial herb belonging to the family *Convolvulaceae* with a small woody and branched root stock[6]. This plant is used in traditional medicine to cure fever, cough, cold, venereal diseases, azoospermia, adenitis and dementia in East Asia, India, Africa and the Philippines. It has a known nootropic and anti-inflammatory activity[7],[8]. The aim of this study was to establish a phytochemical screening and HPTLC finger printing profile of the whole plant ethanolic extract of *E. alsinoides*, which may be used as a marker for quality evaluation and standardization of the drug.

## MATERIALS AND METHODS

### Preparation of plant extract

*E. alsinoides* (L.) L. used in this study was obtained from Coimbatore district, Tamilnadu, India. The plant was authenticated by Dr. P.Satyanarayana, Botanical Survey of India, TNAU Campus, Coimbatore. The voucher number is BSI/SRC/5/23/2011-12/Tech.-514. Fresh whole plant material of *E. alsinoides* was washed under running tap water, air dried and powdered in electric blender. Twenty grams of powdered plant material were mixed with 100 mL of various solvents (petroleum ether, chloroform, ethyl acetate, ethanol and distilled water). The plant extracts were prepared by using soxhlet extraction and an orbitory shaker apparatus. After extraction the samples were collected and stored in a vial for further studies.

### Phytochemical screening

#### Qualitative estimation of phytoconstituents

Phytochemical screening was carried out to assess the qualitative chemical composition of crude extracts and to identify the major natural chemical groups such as steroids, reducing sugars, alkaloids, phenolic compounds, saponins, tannins, flavonoids, amino acids, terpenoids and cardioglycosides using commonly employed precipitation and coloration. General reactions in these analyses revealed the presence or absence of these compounds in the crude extracts tested[9],[10].

#### Quantitative estimation of phytoconstituents

Estimation of carbohydrate: The total amount of carbohydrates present in the ethanolic extract of *E. alsinoides* was determined by the standard method given by Sadasivam and Manickam[11].

Estimation of total phenols: Total phenolic content of the ethanolic extract of *E. alsinoides* was measured based on the Folin-Ciocalteu assay[12]. Briefly, 0.5 mL of the ethanolic extract was first mixed with 2.5 mL of distilled water, and then 0.5 mL of Folin-ciocalteu reagent was added. After 3 min, 2 mL of 20% sodium carbonate was added and mixed thoroughly. The tubes were incubated in a boiling water bath for exactly 1 min. It was then cooled and the absorbance was measured at 650 nm using a spectrophotometer against the reagent blank. Total phenolic content was expressed as mg gallic acid equivalents (GAE)/g fresh weight.

Estimation of total flavonoids: The flavonoid content was examined by adopting the method developed by Ordon *et al*.,[13]. Briefly, 0.5 mL of 2% AlCl_3_ in ethanol solution was added to 0.5 mL of sample solution. After one h incubation at room temperature, yellow colour was developed. This was measured at 420 nm with a UV-visible spectrophotometer. A standard graph was prepared using the quercetin and the total flavonoid content was expressed as quercetin equivalent (mg/g).

Estimation of tannins: The tannin content was estimated by the Vanillin-HCL method[11]. Briefly, 0.5 mL of distilled water and 4.0 mL of Vanillin-HCL were added to 0.5 mL of sample and mixed thoroughly. The absorbance of all samples was measured at 500 nm using a spectrophotometer. The amount of tannin present in the sample was calculated from the standard graph.

### HPTLC analysis

Two µL of the above test solution and 2 µL of standard solution were loaded as 5 mm band length in the 3×10 Silica gel 60F254 TLC plate using a Hamilton syringe and CAMAG LINOMAT 5 instrument. The samples-loaded plate was kept in TLC twin trough developing chamber (after saturation with solvent vapor) with respective mobile phases (alkaloids, flavonoids and phenols) and the plate was developed in the respective mobile phase up to 90 mm. The developed plate was dried by hot air to evaporate solvents from the plate. The plate was kept in a photo-documentation chamber (CAMAG REPROSTAR 3) and the images were captured at white light, UV 254 nm and UV 366 nm. The developed plate was sprayed with respective spray reagents (alkaloids, flavonoids and phenols) and dried at 100°C in a hot air oven. The plate was photo-documented at daylight and UV 366 nm mode using a photo-documentation (CAMAG REPROSTAR 3) chamber. After derivatization, the plate was fixed and scanning was done at 500 nm by TLC Scanner 3. The Peak table, Peak display and Peak densitogram were examined.

**Table 1:** Phytochemical analysis of *Evolvulus alsinoides* (L.)L.

Phytochemical constituents	Solvent				
	Petroleumether	Chloroform	Ethyl acetate	Ethanol	Water
Alkaloids	-	-	-	+	+
Steroids	+	+	+	+	-
Flavonoids	-	-	-	+	+
Tannins/phenols	-	+	-	+	-
Aminoacids and proteins	-	-	+	+	+
Sugars	-	+	+	+	+
Cardioglycosides	+	+	+	+	-
Saponins	-	-	-	-	-
Terpenoids	-	-	-	+	+

“+” indicates presence of secondary metabolites; “-” indicates absence of secondary metabolites.

**Table 2:** Quantitative estimation of phytoconstituents

Phytoconstituents	Ethanolic extract of E. alsinoides (L.)L.
Carbohydrate (mg/g)	7.3±0.268
Tannin (mg/g)	16.0±0.894
Phenol (mg/g)	192.0±0.900
Flavonoid (mg/g)	26.0±0.516

(mean±SD)

#### For alkaloids

Mobile phase: Ethyl acetate-methanol-water (10: 1.35:1). Spray reagent: Dragendorff's reagent followed by 10% ethanolic sulphuric acid reagent. Detection: Yellow-brown coloured zones at day light mode present in the given standard and sample track observed in the chromatogram after derivatization, which confirmed the presence of alkaloids in the given standard and in the sample.

#### For flavonoids

Mobile phase: Ethyl acetate-butanone-formic acid-water (5:3:1:1). Spray reagent: 1% ethanolic aluminium chloride reagent. Detection: Yellow coloured fluorescent zone at UV 366 nm mode present in the given standard and sample track observed in the chromatogram after derivatization; which confirmed the presence of flavonoid in the given standard and in the sample.

#### For phenol

Mobile phase: Toluene-acetone-formic acid (4.5: 4.5:1). Spray reagent: 20% sodium carbonate solution followed by Folin Cio-calteu reagent. Detection: Blue coloured zones at daylight mode observed in the chromatogram after derivatization, confirmed the presence of phenolic compound in the given standard and in the sample.

## RESULTS

Phytochemical screening helps to reveal the chemical nature of the constituents of the plant extract and the one that predominates over the others. It may also be used to search for bioactive agents that could be used in the synthesis of very useful drugs[5],[14]. The bioactive compounds present in *E. alsinoides* were qualitatively analyzed and the results revealed that most of secondary metabolites were present in the ethanolic extract of *E. alsinoides* (L.)L. (***Table 1***).

The total carbohydrate, phenols, and flavonoid content of the ethanolic extract of *E. alsinoides* were studied. Total carbohydrate, tannin, phenol and flavanoid contents were found to be 7.3 mg/g, 16.00 mg/g, 192 mg/g and 26 mg/g, respectively (***Table 2***).

**Table 3:** Peak table with Rf values, height and area of alkaloids and unknown compounds.

Track	Peak	Rf	Height	Area	Assigned substance
COL	1	0.41	132.8	3583.7	Colchicine standard
Sample A	1	0.10	61.2	1604.4	Unknown
Sample A	2	0.19	436.2	30400.6	Alkaloid 1
Sample A	3	0.27	168.9	5307.9	Alkaloid 2
Sample A	4	0.35	57.3	1522.7	Alkaloid 3
Sample A	5	0.45	47.7	1608.3	Alkaloid 4
Sample A	6	0.56	38.5	444.2	Unknown
Sample A	7	0.61	149.8	3985.5	Alkaloid 5
Sample A	8	0.75	25.5	210.4	Unknown
Sample A	9	0.83	39.2	1159.7	Unknown
Sample A	10	0.90	445.1	31614.6	Unknown

Rf: retardation factor; COL: colchicine.

**Fig. 1:**
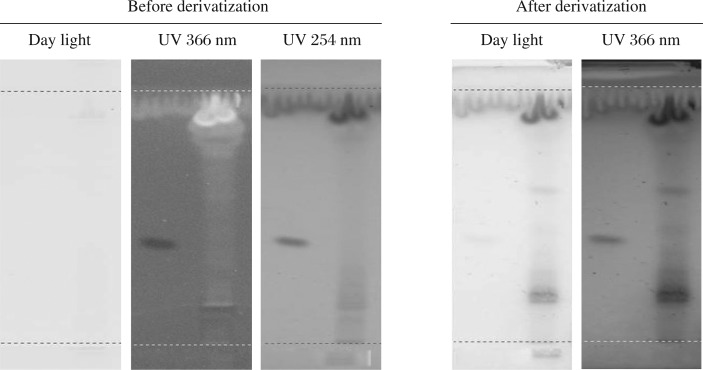
HPTLC Chromatogram of ethanolic extract of *Evolvulus alsinoides* (L.) L. Bands were observed in the chromatogram after derivatization confirmed the presence of alkaloids. Before derivatization: under daylight, under UV 254 nm, under UV 366 nm. After derivatization: under daylight and UV 366 nm.

**Fig. 2:**
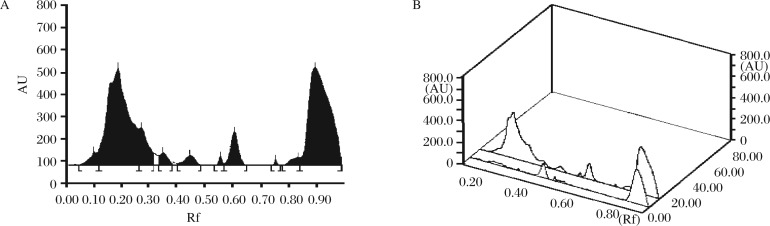
Densitogram (A) and 3D (B) display for alkaloids of the ethanolic extract of *Evolvulus alsinoides*. HPTLC chromatogram of the ethanolic extract at 500 nm, showing different peaks (bands) of phytoconstituents of *Evolvulus alsinoides* (L.) L. Rf: retardation factor.

**Table 4:** Peak table with Rf values, height and area of flavonoids and unknown compounds

Track	Peak	Rf	Height	Area	Assigned substance
RUT	1	0.49	403.2	14734.5	Rutin standard
Sample A	1	0.06	10.3	103.2	Unknown
Sample A	2	0.11	12.8	231.0	Unknown
Sample A	3	0.16	15.7	335.1	Unknown
Sample A	4	0.24	153.0	5608.0	Flavonoid 1
Sample A	5	0.30	211.5	7791.7	Flavonoid 2
Sample A	6	0.42	35.8	1551.5	Flavonoid 3
Sample A	7	0.48	60.3	2015.2	Flavonoid 4
Sample A	8	0.58	15.1	349.0	Unknown
Sample A	9	0.65	82.2	2672.5	Flavonoid 5
Sample A	10	0.75	72.2	2399.4	Flavonoid 6
Sample A	11	0.94	118.6	2423.4	Unknown
Sample A	12	0.96	191.1	5089.3	Unknown

Rf: retardation factor; RUT: rutin.

**Fig. 3:**
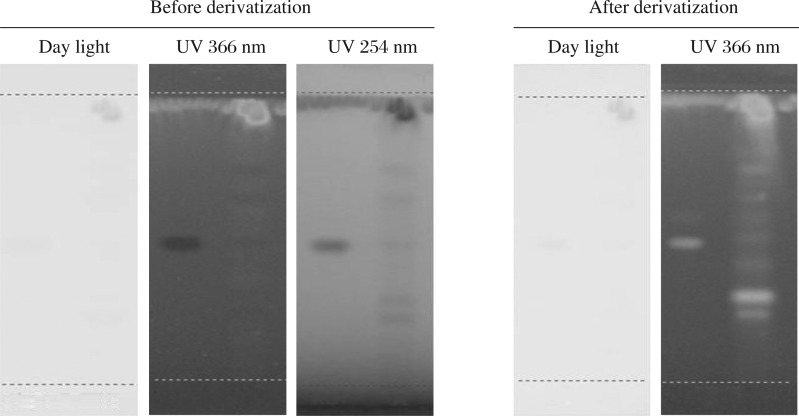
HPTLC studies on the flavonoids of ethanolic extract of *Evolvulus alsinoides* (L.) L. Fluorescent zone at UV 366 nm mode was observed in the chromatogram after derivatization confirmed the presence of flavonoids. Before derivatization: under day light, UV 254 nm and UV 366 nm. After derivatization: under day light and UV 366 nm.

HPTLC profile of the ethanolic extract of *E. alsinoides* is shown in ***Table 3***, ***Table 4*** and ***Table 5*** for alkaloids, flavonoids and phenols; respectively. Yellow-brown coloured zones were detected in daylight and UV after derivatization for alkaloid, yellow coloured fluorescent zone at UV 366 nm for flavonoid and blue coloured zones for phenols in the chromatogram. The ethanolic extract was run along with the standard alkaloid, flavonoid and phenolic compounds. The Rf values of the plant extract were found to be 0.19, 0.27, 0.35, 0.45, 0.61 of peak 2, 3, 4, 5, and 7, respectively. Among them, peaks 2, 3, 4, 5 and 7 were found as alkaloids. The peak height of the respective alkaloids is also given in ***Table 3***, ***Fig. 1*** and ***Fig. 2***.

**Fig. 4:**
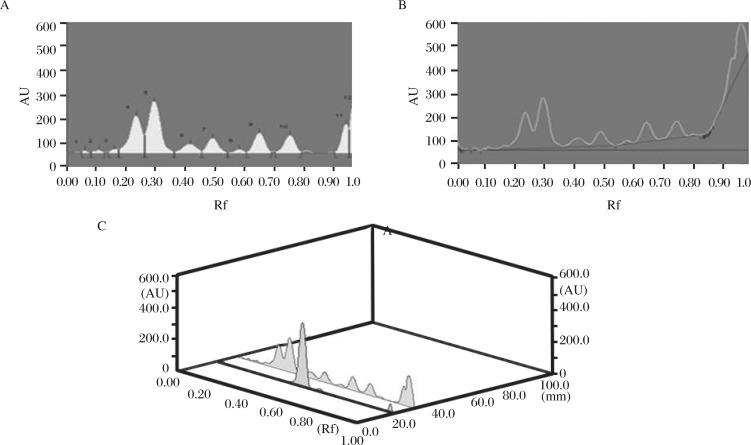
Densitogram (A) baseline (B) and 3D (C) display of flavonoid of the ethanolic extract of *Evolvulus. alsinoides*. HPTLC chromatograms of the ethanolic extract at 366 nm, showing different peaks (bands) of phytoconstituents in *Evolvulus. alsinoides* (L.)L. The concentration of the samples was 100 mg/mL. Ethyl acetate-butanone-formic acid-water (5: 3: 1: 1). Rf: retardation factor.

**Fig. 5:**
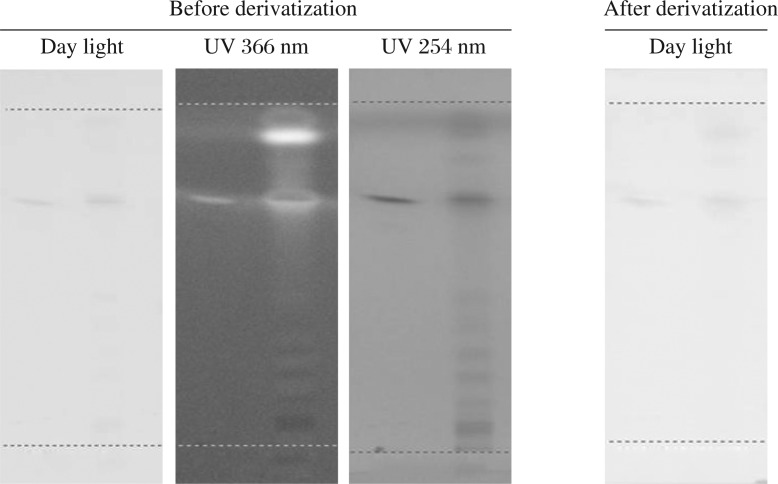
Photo documentation of ethanolic extract of *Evolvulus alsinoides* (L.) L. Zones at day light mode was observed in the chromatogram confirmed the presence of phenolic compounds. Chromatograms of extracts in HPTLC analysis. Before derivatization: under day light, under UV 254 nm, and under UV 366 nm.

**Fig. 6:**
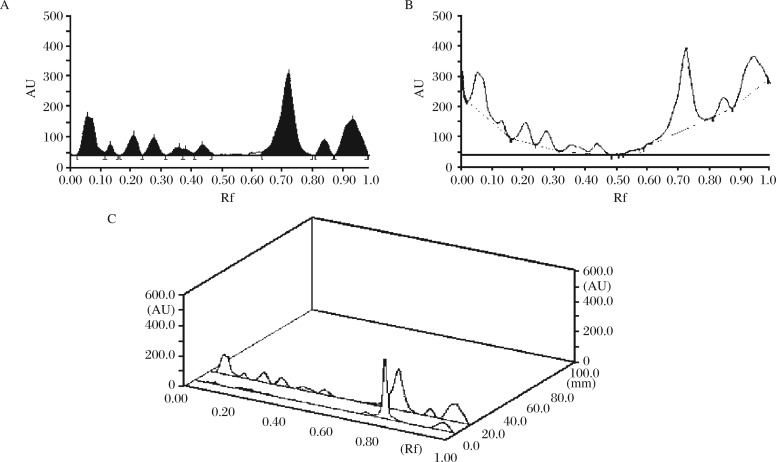
Densitogram (A), baseline (B) and 3D (C) display of phenols of the ethanolic extract of *Evolvulus alsinoides*. HPTLC chromatogram of the ethanolic extract of *Evolvulus alsinoides* at 254 nm, showing different peaks (bands) of phytoconstituents in which the 8th peak was found to be quercetin with a retardation factor (Rf) value of 0.73.

***Table 4*** shows the Rf value of plant extract for flavonoid in which the peaks of 4, 5, 6, 7, 9 and 10 were found as flavonoids (***Fig. 3*** and ***4***). The peak height of the respective flavonoids is also given in ***Table 4***. The phenolic compound showed the Rf values of 0.06, 0.13, 0.21, 0.28, 0.36, 0.38, 0.44, 0.73, 0.84 and 0.94 with the peak of 1 – 10. Among them, the 8, 9 and 10^th^ peaks were found as phenols. The 8^th^ peak showed the presence of quercetin in our samples. The peak height of the respective phenolic compounds is given in ***Table 4***, ***Fig. 5*** and ***Fig. 6***.

**Table 5:** Peak table with Rf values, height and area of phenols and unknown compounds

Track	Peak	Rf	Height	Area	Assigned substance
QUE	1	0.73	392.4	7334.5	Quercetin standard
Sample A	1	0.06	127.6	4516.8	Unknown
Sample A	2	0.13	33.5	582.9	Unknown
Sample A	3	0.21	65.0	1759.3	Unknown
Sample A	4	0.28	55.0	1504.7	Unknown
Sample A	5	0.36	25.5	633.8	Unknown
Sample A	6	0.38	20.9	398.3	Unknown
Sample A	7	0.44	34.4	924.8	Unknown
Sample A	8	0.73	269.9	11278.3	Phenolic 1 (Quercetin)
Sample A	9	0.84	49.8	1223.2	Phenolic 2
Sample A	10	0.94	117.9	5970.9	Phenolic 3

Rf: retardation factor; QUE: quercetin.

## DISCUSSION

Phytomedicines have been used for the treatment of diseases as done in cases of Unani and Ayer Vedic system of medicines, a natural blueprint for the development of new drugs. Much of the exploration and utilization of natural products as antimicrobials arises from microbial sources[15]. This study was to analyze phytochemical screening and HPTLC finger printing analysis of whole plant ethanolic extract of *E. alsinoides* (L.)L.

Knowledge of the phytochemical constituents of plants is desirable, not only for the discovery of therapeutic agents, but also for the discovery of new economic materials such as tannins, oils, gums, flavonoids, saponins, precursors for the synthesis of complex chemical substances[16]. The preliminary phytochemical screening carried out by Omogbai and Eze showed that *E. alsinoides* contains some secondary metabolites such as glycosides, alkaloids, poly phenols, carbohydrates, amino acids and proteins, saponins, volatile oil, flavonoids and tannins[8].

Plant essentials or volatile oils and their individual components have been used in traditional medicine against a variety of bacterial infections for centuries. Furthermore, it has been demonstrated that antibacterial properties of these oils can be attributed to their hydrocarbon and terpene constituents[17]. The results of this research highlighted the fact that ethanol extracted the most phytochemical constituents. This observation agreed with previous reports of medicinal plants that organic solvents were more suitable for extraction of phytochemicals[18],[19].

The carbohydrates produced by plants are found to be an important source of energy for animals. Phenolic antioxidants are potent free radical terminators[20]. They donate hydrogen to free radicals and hence break the reaction of lipid peroxidation at the initiation step[21]. The high potential of phenolic to scavenge free radicals may be due to its many hydroxyl groups[22].

HPTLC is useful as a phytochemical marker and also a good estimator of genetic variability in plant populations. The presence or absence of chemical constituent has been found useful in the placement of the plant in taxonomic categories. HPTLC profile differentiation is such an important and powerful procedure which is often employed for this purpose. HPTLC fingerprinting is proved to be a liner, precise, accurate method for herbal identification and can be used further in authentication and characterization of the medicinally important plant. The developed HPTLC fingerprints will help the manufacturer for quality control and standardization of herbal formulations[23].

In conlusion, the medicinal plant extracts could be an answer to people seeking for better therapeutic agents from natural sources, which are believed to be more efficient with little or no side effects when compared to the commonly used synthetic chemotherapeutic agents. Based on the results of the study, we concluded that the ethanolic extracts of *Evolvulus alsinoides* (L.)L. have a significant amount of secondary metabolites. These metabolites are beneficial for maintenance of human health and chronic degenerative diseases. This might act as a pharmacotherapeutic agent in future and further study is needed for structural elucidation and characterization of bioefficacies of these active compounds.
